# 937. A Pediatric Scar Questionnaire Identifies Patients Who Benefit from Referral to the Burn Reconstruction Team

**DOI:** 10.1093/jbcr/irag033.504

**Published:** 2026-04-06

**Authors:** Kati Venable, Emily Webb, Amanda Ehrhardt, Deborah Knight, Rajiv S Sood

**Affiliations:** Joseph M. Still Burn Center at Doctors Hospital, Augusta, GA; Joseph M. Still Burn Center at Doctors Hospital, Augusta, GA; Joseph M. Still Burn Center at Doctors Hospital, Augusta, GA; Joseph M. Still Burn Center at Doctors Hospital, Augusta, GA; Burn and Reconstructive Centers of America, Augusta, GA

## Abstract

**Introduction:**

In 2023, our ABA verified, outpatient burn clinic completed 3276 pediatric visits. In preparation for reverification, burn clinic leadership recognized the need for on-going quality improvement (QI) in pediatric specific rehabilitation issues, specifically timely access and referral to reconstructive surgery. To address this, a QI project was developed to increase the identification of pediatric burn patients who could benefit from a referral with the burn reconstruction team.

**Methods:**

In January 2024, a pediatric burn scar questionnaire (PBSQ) was developed to identify patients who could benefit from a referral to the burn reconstruction team. Concurrently, an intervention form was developed to track scar management interventions provided. From February 2024 through August 2025, the pediatric burn scar patients or their parent/guardian completed the PBSQ, and the therapist completed the intervention form during the clinic visit. The responses of the PBSQ and intervention form help to identify potential referrals to the burn reconstruction team. The PBSQs were reviewed monthly, and findings were reported at both the monthly burn quality meeting and the bi-monthly burn operations meetings to identify timely referral of pediatric patients to the burn reconstruction team.

**Results:**

There were 981 total PBSQs completed and 669 who received a therapy consult on the same visit. 188 of the PBSQs were completed by active pediatric patients of the burn reconstruction team. 137 participants were identified as potential candidates for referral to the burn reconstruction team. 38 patients received a referral to the burn reconstruction team. The highest percentage (35.14%) of burn scar patients identified to benefit from a referral to the reconstruction team was in December 2024. The lowest percentage (2.94%) was April 2025. The two most frequent therapy interventions provided to the patients were interim compression garments and scar massage education. The two most frequent scar assessment measures used by therapists were the Modified Vancouver Scar Scale and the Itch Man Scale for Pruritus.

**Conclusions:**

By surveying pediatric patients or their parent/guardian on their burn scar management we were able capture chronic scar conditions and sequalae requiring reconstructive intervention. Inconsistencies in the referral of pediatric patients to the reconstruction team are ongoing but showing improvement as a direct result of tracking and reporting our findings. Timely referral to the reconstruction team ensures pediatric patients receive all necessary scar management interventions and ongoing assessment.

**Applicability of Research to Practice:**

Development of standardized referral criteria to the reconstruction team prevents discrepancy of practice following interdisciplinary turnover. By asking specific questions we ensure comprehensive interventions and ongoing assessment of pediatric patients who experience the chronic condition of burn injuries.

**Funding for the study:**

N/A.

